# Factors influencing smoking cessation counselors' intention to stay: An application of a conceptual model of intention to stay verified with path analysis

**DOI:** 10.3389/fpubh.2022.932213

**Published:** 2022-09-20

**Authors:** Yi-Chun Liu, Shih-Hung Chiang, Chung-Yu Lai, Li-Chen Yen, Fang-Yih Liaw, Ming-Han Lin, Fu-Gong Lin, Ching-Huang Lai, Senyeong Kao, Yu-Tien Chang, Chia-Chao Wu, Yu-Lung Chiu

**Affiliations:** ^1^Graduate Institute of Medical Sciences, National Defense Medical Center, Taipei, Taiwan; ^2^School of Public Health, National Defense Medical Center, Taipei, Taiwan; ^3^Graduate Institute of Aerospace and Undersea Medicine, National Defense Medical Center, Taipei, Taiwan; ^4^Department of Microbiology and Immunology, National Defense Medical Center, Taipei, Taiwan; ^5^Department of Family and Community Medicine, Tri-Service General Hospital, Taipei, Taiwan; ^6^Graduate Institute of Life Sciences, National Defense Medical Center, Taipei, Taiwan; ^7^Division of Nephrology, Department of Internal Medicine, Tri-Service General Hospital, National Defense Medical Center, Taipei, Taiwan

**Keywords:** smoking cessation, counselor, intention to stay, conceptual model of intent to stay, path analysis

## Abstract

**Background:**

The Taiwanese military trains smoking cessation counselors to counsel officers and soldiers on quitting smoking as part time. The intention to stay among smoking cessation counselors affects the promotion of smoking cessation. This study investigated smoking cessation counselors' intention to stay by applying a conceptual model of intent to stay (CMIS) to analyze influencing factors.

**Methods:**

In this cross-sectional study, we applied the CMIS to design a questionnaire. We invited 577 smoking cessation counselors trained in the military from 2016 to 2017. The response rate was 46.7%, and the questionnaire responses of 260 military smoking cessation counselors were analyzed. We used path analysis to verify the relationships among the various aspects of the CMIS.

**Results:**

We determined that smoking cessation counselors' intention to stay is directly affected by job satisfaction (β = 0.150, *p* = 0.014), job stress (β = −0.225, *p* < 0.001), and institutional identification (β = 0.431, *p* < 0.001). Career opportunities indirectly affect intention to stay through institutional identification, working environment indirectly affects intention to stay through job stress, and co-worker support and self-fulfillment indirectly affect intention to stay through job satisfaction and institutional identification. Our model could explain 36.7% of the variance in intent to stay among smoking cessation counselors.

**Conclusion:**

Our results suggest that relevant policies should be formulated to enhance smoking cessation counselors' recognition, affirmation, and sense of belonging as related to smoking cessation counseling work, thereby raising their institutional identification and promoting their intention to stay.

## Introduction

Smoking is notoriously harmful to health. According to the WHO, half of the deaths of people who smoke regularly can be attributed to their smoking habits. Globally, smoking-related diseases are the second leading cause of death, and smoking is also the fourth most common risk factor for disease. Approximately, 6 million adults die from smoking-related diseases each year (1).

According to a survey, the prevalence of cigarette smoking among young conscripts in military training centers and military officers in various services was 31.0–36.7% in Taiwan in 2014, which were considerably higher than Taiwan's national smoking rate and the U.S. army's smoking rate in the same year (16.4 and 13.9%, respectively) (2–4). The prevention and control of smoking in the Taiwan military must be implemented to address this trend.

In Taiwan, smoking cessation counselors are military personnel who were assigned by their units to receive training and to help officers and soldiers cope with the military mission and the particularity of the military workplace environment, maintain the health of them, increase the effectiveness of smoking cessation services and resources provided to basic units of the military, and encourage officers and soldiers to quit smoking, thereby reducing the military smoking rate. The Medical Affairs Bureau of the Ministry of National Defense manages the training of tobacco hazard prevention and control counselors. The officers, noncommissioned officers, soldiers, or others who intend to engage in smoking prevention and control were assigned by their unit to join the smoking cessation counselors training. An average of 300 smoking cessation counselors are trained each year. Four sessions are conducted in northern, central, southern, and eastern Taiwan annually, with free participation for tobacco hazard prevention and control counselors of all military units, psychological counseling staff, emergency medical technicians, and officers and soldiers who intend to engage in smoking prevention and control. The 3-h training courses cover awareness of smoking hazards and the relevance of smoking diseases, how to prompt motivation to quit, smoking cessation counseling and counseling skills, and other topics. Participants receive a smoking cessation counselor's training certificate if they pass the written test with a score of at least 80 points. Each military smoking cessation counselor serves officers and soldiers, assisting them with the task of quitting smoking through social support methods such as companionship, assistance, counseling, consultation, and referral (5). In order to assist military smoking cessation counselors in adjusting their coaching implementation, such as caring, companionship, assistance, counseling, or consultation, families of military smoking cessation counselors have been established, either through smoking cessation doctors in military hospitals or in grassroots troops serving as instructors. Moreover, military smoking cessation counselors can also refer smokers to specialists in military hospitals who can provide medication to help them quitting. Additionally, groups of military smoking cessation counselors discussed their work and shared their experiences, forming beneficial cycles (6).

A meta-analysis of 52 articles on the effectiveness of counseling and medications for smoking cessation revealed that the effect of medication and counseling on smoking cessation is stronger than that of general care [relative risk (RR) = 1.83] regardless of whether the treatment occurs in medical care institutions (RR = 1.97) or communities (RR = 1.53) (7). Because counseling can help people quit smoking more effectively than can other methods, the Ministry of National Defense has been training smoking cessation counselors every year since 2011 to enhance the military's smoking cessation rate because insufficient military medical officers are available to support a large number of officers and soldiers undergoing smoking cessation. Providing care, companionship, consultation, and referrals to the National Military Hospital are all part of a responsibilities of smoking cessation counselor. However, smoking cessation counseling is a part-time endeavor, and each counselor has a full-time job in the military. The willingness of counselors to continue counseling work affects the military's smoke prevention and control efforts.

Studies have reported that intention to stay is a predictor of resignation behavior (8). Numerous factors influence an employee's intention to stay. In 1999, Boyle et al. proposed a conceptual model of intent to stay (CMIS) to analyze intention to stay among nurses. In this theoretical framework, manager, organizational, nurse, and work characteristics influence individuals' intention to stay through factors, such as job satisfaction, job stress, and commitment. The overall explanatory power of the model is 52% (9). The model was also applied in a Swiss study of the factors that influence the intention to stay of various medical care employees in hospitals (10). However, previous studies have focused solely on full-time employment, and no studies have yet applied the model to analyze the intention to stay of part-time employees. Thus, this study investigated whether the CMIS can be used to predict and explore the factors affecting smoking cessation counselors' intention to stay.

## Materials and methods

### Study design and participants

Participants were not involved in the design or conduct of this study. The experiment design and procedures of this study were performed according to the guidelines of the Declaration of Helsinki. For this cross-sectional study, the inclusion criteria were smoking cessation counselors who have attended a training course and received training certificates conducted by the Medical Affairs Bureau of the Ministry of National Defense in 2016–2017. The total number of smoking cessation counselors was 740. After the telephone interview, 29 counselors were veterans and 154 counselors were with missing phone number information; thus they were excluded. Finally, 577 counselors completed the survey.

### Measurements

We contacted qualified counselors over the phone to request their participation in this study. After obtaining their consent, we emailed the online version of the questionnaire to the addresses provided by the counselors. The participants completed the questionnaire and submitted their responses online.

The questionnaire was developed with reference to relevant literature, which comprised basic characteristics, the CMIS (24 items gathered in eight dimensions: manager characteristics, workload, career opportunities, working environment, work organization, coworker support, self-fulfillment, and institutional identification), a job stress scale (six items), a job satisfaction scale (10 items), and a single item regarding intention to stay (10–13).

The counselors' basic characteristics included age, sex, educational attainment [senior high school, vocational high school, junior college, university of science and technology (including institute of technology), college, or graduate institute], higher education major (medical-related or nonmedical related), military service branch (army, navy, air force, military police, reserve force, and affiliated units of the Ministry of National Defense), military rank (officer, noncommissioned officer, soldier, or other), years of service, years of service as counselor, position (leadership or nonleadership), professional specialty [military doctor (including military health service) or nonmilitary doctor], location of military station (northern, central, southern, eastern, or outer islands), and workplace category (grassroots troop or nongrassroots troop).

The items on the questionnaire employed in this study were developed according to the CMIS. Face validity of the questionnaire was reviewed by health-care and health-care education experts. After several revisions, the content validity index of the questionnaire was determined to be 0.97.

A four-point scale was adopted for CMIS items (1 = strongly disagree to 4 = strongly agree). CMIS items included manager characteristics (seven items), workload (three items), career opportunities (two items), working environment (three items), work organization (two items), coworker support (two items), self-fulfillment (two items), and institutional identification (three items). Items of manager characteristics were as follows: (1) your manager is available when you need his/her help to perform counseling work, (2) your manager appreciates your smoking cessation counseling work, (3) your manager recognizes your competences as a counselor, (4) your manager supervises you sufficiently when you perform counseling work, (5) your manager is respectful with his/her members of the counseling team, (6) your manager behaves fairly with all members of the counseling team fairly, and (7) your manager leads and motivates his team members satisfactorily. The average score of the seven items was obtained, and Cronbach's α was 0.959. Items of workload were as follows: (1) you are able to accomplish your counseling work within the scheduled time, (2) the distribution of the workload to members is with equity in your counseling team, and (3) your work situation as a counselor (e.g., schedules, holidays, leisure) allow work and private life combination. The average score of these three items was obtained, and Cronbach's α was 0.872. Items of career opportunities were as follows: (1) your superiors encouraged your professional development as a counselor, and (2) in your opinion, counseling is a career perspective possible for you in the institution (e.g., promotion, mobility)? The average score of these two items was obtained, and Cronbach's α was 0.823. Items of working environment were as follows: (1) the organization climate well-suited to your work demands of counseling work, (2) the facilities and equipment in your working conditions well-suited to your work demands of counseling work, and (3) smoking cessation medications and other related consumables in your working conditions well-suited to your work demands of counseling work. The average score of these three items was obtained, and Cronbach's α was 0.885. Items of work organization were as follows: (1) globally, the work of counselors in your organization is well-organized, and (2) the information sharing regarding counseling work in your organization is well-organized. The average score of these two items was obtained, and Cronbach's α was 0.949. Items of coworker support were as follows: (1) you can count on your colleagues' support regarding counseling work, and (2) the relationships among counselors in your organization are respectful. The average score of these two items was obtained, and Cronbach's α was 0.904. Items of self-fulfillment were as follows: (1) when working as a counselor, you have the occasion to use your skills and abilities, and (2) you enjoy coming to work as a counselor. The average score of these two items was obtained, and Cronbach's α was 0.905. Items of institutional identification were as follows: (1) you are proud to serve as a counselor, (2) you share the idea conveyed in counseling work with others, and (3) the work of counselors contributes to the execution of the military's smoking cessation efforts. The average score of these three items was obtained, and Cronbach's α was 0.895. Cronbach's α of CMIS items are shown in Table 1.

**Table 1:** Cronbach's α of CMIS items.

**CMIS items**	**Items**	**Cronbach's α**
Manager characteristics	7	0.959
Workload	3	0.872
Career opportunities	2	0.823
Working environment	3	0.885
Work organization	2	0.949
Co-worker support	2	0.904
Self-fulfillment	2	0.905
Institutional identification	3	0.895

A four-point scale was also adopted for job stress items (1 = strongly disagree to 4 = strongly agree). In contrast to the general scale, a higher job stress score indicated stronger disagreement. Items of job stress were as follows: (1) counseling work is physically demanding, (2) you are under constant time pressure due to a heavy workload associated with counseling, (3) you have very little freedom to decide how to do counseling work, (4) considering the heavy workload associated with counseling, you have to work very fast, (5) you often feel bothered or upset when working as a counselor, and (6) the demands of Counseling work interfere with your personal life. The average score of these six items was obtained, and Cronbach's α was 0.944.

A five-point Likert scale was adopted for job satisfaction items (1 = strongly dissatisfied to 5 = strongly satisfied). Items of job satisfaction were as follows: (1) autonomy associated with counseling work, (2) challenges associated with counseling work, (3) responsibilities associated with counseling work, (4) opportunities for advancement associated with counseling work, (5) occupational image associated with counseling work, (6) creativity associated with counseling work, (7) job security associated with counseling work, (8) professional growth associated with counseling work, (9) nature of work associated with counseling work, and (10) content of work associated with counseling work. The average score of these ten items was obtained, and Cronbach's α was 0.964.

With all reliability scores (Cronbach's α values) over 0.7, the scale had high reliability (14).

For intention to stay, only one item was listed: intention to stay as a smoking cessation counselor. The single item regarding intention to stay was scored on a 7-point scale (1 = strongly unwilling to 7 = strongly willing).

### Data analysis

We employed SPSS 22.0 (IBM, Armonk, NY, USA) for statistical analysis. Continuous variables are expressed as means and standard deviations, and categorical variables are expressed as frequencies and percentages. Independent *t*-tests, one-way analysis of variance, and a Pearson correlation test were used to analyze the relationships among demographic characteristics, job satisfaction, job stress, institutional identification, and the intention to stay.

The CMIS was evaluated through path analysis using Amos 25.0 modeling software (IBM SPSS). The goodness-of-fit indices for the model were as follows: the *p*-value of the chi-square (χ^2^) test was >0.05, the value of the relative χ^2^ (χ^2^/degrees of freedom) was <3, the root mean square error of approximation was < 0.05, goodness-of-fit index was >0.90, and normed fit index was >0.90 (15).

## Results

### Demographics

Of the 557 counselors initially screened, 260 were included in the analysis. The response rate was 46.7%. The demographic characteristics of the study population are presented in Table 2. The average age of participants was 30.46 years, and the proportion of male participants (75.0%) was higher than that of female participants (25.0%). The most common level of education was college (39.0%), and most participants had majors that were not medical related (61.2%). Nearly half the participants served in the army (*n* = 127, 49.8%), 22.4% (*n* = 57) in the navy, 17.6% (*n* = 45) in the air force, and 10.2% (*n* = 26) in other military service branches. The most common military rank among the participants was noncommissioned officer (45.7%). The participants' average length of military service was 7.33 years, and the average length of service as a counselor was 1.91 years. Most participants were in nonleadership positions (71.9%) and were part of the military medical service (56.5%). The most common location of the military station was the south (30.0%). Most participants worked in grassroots troops (78.5%).

**Table 2:** Demographic characteristics of study population.

**Variables**	***n* (%)/Mean ±SD**
Age	30.46 ± 6.10
**Sex**
Male	192 (75.0)
Female	64 (25.0)
**Educational attainment**
Senior high school	16 (6.3)
Vocational high school	45 (17.7)
Junior college	45 (17.7)
University of Science and Technology	39 (15.4)
College	99 (39.0)
Graduate institute	10 (3.9)
**Higher education major**
Medical-related	93 (38.8)
Nonmedical related	147 (61.2)
**Military service branch**
Army	127 (49.8)
Navy	57 (22.4)
Air Force	45 (17.6)
Military Police	12 (4.7)
Reserve force	10 (3.9)
Affiliated units of the Ministry of National Defense	4 (1.6)
**Military rank**
Officer	74 (28.9)
Noncommissioned officer	117 (45.7)
Soldier	61 (23.8)
Others	4 (1.6)
Years of service	7.33 ± 6.16
Years of service as counselor	1.91 ± 1.27
**Position**
Leadership	73 (28.1)
Nonleadership	187 (71.9)
**Professional specialty**
Military doctor	135 (56.5)
Nonmilitary doctor	104 (43.5)
**Location of military station**
Northern	71 (27.3)
Central	41 (15.8)
Southern	78 (30.0)
Eastern	24 (9.2)
Outer islands	46 (17.7)
**Workplace category**
Grassroots troop	56 (21.5)
Nongrassroots troop	204 (78.5)

SD, Standard deviation.

### Descriptive statistics for the CMIS

The distribution of the CMIS dimension scores is presented in Table 3. The mean scores for seven dimensions of the CMIS (average scores 2.62–2.92 points), for job satisfaction (3.24 points), and for intention to stay (4.64 points) were above the scale midpoint. The results revealed that the smoking cessation counselors had a positive opinion regarding manager characteristics, career opportunities, working environment, work organization, coworker support, self-fulfillment, institutional identification, job satisfaction, and intention to stay. However, the average workload score was 2.19 points, and the average job stress score was 2.38 points, both of which were below the respective scales' midpoints.

**Table 3:** Descriptive statistics for the effect of CMIS-based questionnaire items on intention to stay.

**Variables**	**Mean ±SD**
**Manager characteristics** ^**a**^	
Your manager is available when you need his/her help to perform counseling work.	2.98 ± 0.57
Your manager appreciates your smoking cessation counseling work.	2.87 ± 0.63
Your manager recognizes your competences as a counselor.	2.92 ± 0.57
Your manager supervises you sufficiently when you perform counseling work.	2.90 ± 0.62
Your manager is respectful with his/her members of the counseling team.	2.95 ± 0.57
Your manager behaves fairly with all members of the counseling team fairly.	2.98 ± 0.57
Your manager leads and motivates his team members satisfactorily.	2.82 ± 0.66
Average score	2.92 ± 0.54
**Workload** ^**a**^	
You are able to accomplish your counseling work within the scheduled time.	2.25 ± 0.58
The distribution of the workload to members is with equity in your counseling team.	2.16 ± 0.55
Your work situation as a counselor (e.g., schedules, holidays, leisure) allow work and private life combination.	2.16 ± 0.57
Average score	2.19 ± 0.50
**Career opportunities** ^**a**^	
Your superiors encouraged your professional development as a counselor.	2.68 ± 0.61
In your opinion, counseling is a career perspective possible for you in the institution (e.g., promotion, mobility)?	2.70 ± 0.67
Average score	2.69 ± 0.59
**Working environment** ^**a**^	
The organization climate well-suited to your work demands of counseling work.	2.71 ± 0.61
The facilities and equipment in your working conditions well-suited to your work demands of counseling work.	2.52 ± 0.72
Smoking cessation medications and other related consumables in your working conditions well-suited to your work demands of counseling work.	2.62 ± 0.70
Average score	2.62 ± 0.61
**Work organization** ^**a**^	
Globally, the work of counselors in your organization is well-organized.	2.70 ± 0.61
The information sharing regarding counseling work in your organization is well-organized.	2.73 ± 0.61
Average score	2.71 ± 0.60
**Co-worker support** ^**a**^	
You can count on your colleagues' support regarding counseling work.	2.90 ± 0.56
The relationships among counselors in your organization are respectful.	2.93 ± 0.54
Average score	2.92 ± 0.53
**Self-fulfillment** ^**a**^	
When working as a counselor, you have the occasion to use your skills and abilities.	2.81 ± 0.58
You enjoy coming to work as a counselor.	2.81 ± 0.58
Average score	2.81 ± 0.55
**Institutional identification** ^**a**^	
You are proud to serve as a counselor.	2.84 ± 0.55
You share the idea conveyed in counseling work with others.	2.90 ± 0.56
The work of counselors contributes to the execution of the military's smoking cessation efforts.	2.93 ± 0.53
Average score	2.89 ± 0.50
**Job stress** ^**a**^	
Counseling work is physically demanding.	2.35 ± 0.72
You are under constant time pressure due to a heavy workload associated with counseling.	2.33 ± 0.71
You have very little freedom to decide how to do counseling work.	2.46 ± 0.73
Considering the heavy workload associated with counseling, you have to work very fast.	2.48 ± 0.71
You often feel bothered or upset when working as a counselor.	2.34 ± 0.73
The demands of Counseling work interfere with your personal life.	2.32 ± 0.75
Average score	2.38 ± 0.64
**Job satisfaction** ^**b**^	
Autonomy associated with counseling work	3.28 ± 0.79
Challenges associated with counseling work	3.29 ± 0.77
Responsibilities associated with counseling work	3.25 ± 0.74
Opportunities for advancement associated with counseling work	3.06 ± 0.78
Occupational image associated with counseling work	3.28 ± 0.77
Creativity associated with counseling work	3.23 ± 0.75
Job security associated with counseling work	3.12 ± 0.75
Professional growth associated with counseling work	3.30 ± 0.75
Nature of work associated with counseling work	3.29 ± 0.77
Content of work associated with counseling work	3.30 ± 0.77
Average score	3.24 ± 0.66
Intention to stay as a smoking cessation counselor^c^	4.64 ± 1.52

^a^1 = strongly disagree to 4 = strongly agree.

^b^1 = strongly dissatisfied to 5 = strongly satisfied.

^c^1 = strongly unwilling to 7 = strongly willing.

SD, standard deviation.

### Univariate analysis

The smoking cessation counselors' characteristics and the results of univariate analysis of job satisfaction, job stress, institutional identification, and intention to stay are presented in Table 4. The male military smoking cessation counselors' average job satisfaction score was significantly higher than that of their female counterparts (3.28 vs. 3.10, *p* = 0.040). Job stress and institutional identification exhibited no significant relationship with the basic characteristics of the smoking cessation counselors. Regarding intention to stay, significant differences in the smoking cessation counselors' intention to stay were identified among counselors with different higher education majors (medical-related or not, 4.91 vs. 4.48, *p* = 0.024), belonging to different professional specialty (military doctor or not, 4.90 vs. 4.38, *p* = 0.011), and with different location of military stations (*p* = 0.038).

**Table 4:** Univariate analysis of the variables.

**Variables**	**Job satisfaction**		**Job stress**		**Institutional identification**		**Intention to stay**	
	***r*/Mean ±SD**	***p*-value**	***r*/Mean ±SD**	***p*-value**	***r*/Mean ±SD**	***p*-value**	***r*/Mean ±SD**	***p*-value**
Age	−0.040	0.528	0.018	0.774	−0.018	0.774	−0.062	0.326
**Sex**		0.040*		0.722		0.924		0.906
Male	3.28 ± 0.69		2.38 ± 0.66		2.89 ± 0.52		4.65 ± 1.54	
Female	3.10 ± 0.56		2.35 ± 0.59		2.89 ± 0.46		4.63 ± 1.48	
**Educational attainment**		0.803		0.393		0.468		0.288
Senior high school	3.11 ± 0.72		2.15 ± 0.61		2.88 ± 0.51		5.00 ± 1.51	
Vocational high school	3.25 ± 0.81		2.46 ± 0.81		2.83 ± 0.63		4.47 ± 1.63	
Junior college	3.18 ± 0.60		2.39 ± 0.63		2.87 ± 0.42		4.42 ± 1.70	
University of Science and Technology	3.18 ± 0.50		2.50 ± 0.51		2.89 ± 0.41		4.56 ± 1.45	
College	3.31 ± 0.64		2.31 ± 0.62		2.90 ± 0.51		4.75 ± 1.44	
Graduate institute	3.30 ± 1.03		2.45 ± 0.53		3.20 ± 0.36		5.50 ± 0.85	
**Higher education major**		0.320		0.935		0.387		0.024*
Medical-related	3.31 ± 0.68		2.38 ± 0.63		2.94 ± 0.47		4.91 ± 1.32	
Nonmedical related	3.22 ± 0.63		2.37 ± 0.64		2.88 ± 0.50		4.48 ± 1.65	
**Military service branch**		0.551		0.500		0.822		0.737
Army	3.21 ± 0.70		2.35 ± 0.66		2.87 ± 0.55		4.75 ± 1.41	
Navy	3.33 ± 0.59		2.37 ± 0.61		2.89 ± 0.46		4.53 ± 1.67	
Air Force	3.28 ± 0.66		2.52 ± 0.68		2.88 ± 0.50		4.47 ± 1.62	
Military Police	3.33 ± 0.48		2.17 ± 0.57		3.06 ± 0.31		4.75 ± 1.29	
Reserve force	3.20 ± 0.62		2.35 ± 0.55		2.83 ± 0.36		4.30 ± 1.83	
Affiliated units of the Ministry of National Defense	2.75 ± 0.54		2.63 ± 0.44		3.08 ± 0.42		4.00 ± 1.41	
**Military rank**		0.669		0.503		0.716		0.057
Officer	3.31 ± 0.66		2.32 ± 0.58		2.92 ± 0.51		5.03 ± 1.34	
Noncommissioned officer	3.19 ± 0.67		2.35 ± 0.67		2.86 ± 0.51		4.50 ± 1.56	
Soldier	3.25 ± 0.68		2.48 ± 0.65		2.90 ± 0.48		4.39 ± 1.62	
Others	3.38 ± 0.56		2.33 ± 0.38		3.08 ± 0.42		5.00 ± 1.63	
Years of service	−0.070	0.259	0.042	0.504	−0.037	0.549	−0.088	0.156
Years of service as counselor	−0.013	0.831	−0.005	0.930	0.005	0.940	−0.039	0.530
**Position**		0.411		0.384		0.468		0.503
Leadership	3.18 ± 0.70		2.32 ± 0.64		2.93 ± 0.50		4.74 ± 1.39	
Nonleadership	3.26 ± 0.65		2.40 ± 0.64		2.88 ± 0.50		4.60 ± 1.57	
**Professional specialty**		0.138		0.687		0.253		0.011*
Military doctor	3.28 ± 0.61		2.37 ± 0.60		2.91 ± 0.45		4.90 ± 1.39	
Nonmilitary doctor	3.15 ± 0.73		2.33 ± 0.69		2.83 ± 0.57		4.38 ± 1.67	
**Location of military station**		0.612		0.447		0.164		0.038*
Northern	3.25 ± 0.64		2.32 ± 0.65		2.91 ± 0.54		4.86 ± 1.56	
Central	3.18 ± 0.74		2.26 ± 0.56		2.76 ± 0.57		4.54 ± 1.29	
Southern	3.26 ± 0.61		2.45 ± 0.59		2.95 ± 0.44		4.58 ± 1.66	
Eastern	3.08 ± 0.66		2.44 ± 0.75		2.75 ± 0.54		3.83 ± 1.20	
Outer islands	3.33 ± 0.73		2.43 ± 0.70		2.96 ± 0.42		4.91 ± 1.43	
**Workplace category**		0.963		0.444		0.566		0.287
Grassroots troop	3.24 ± 0.61		2.44 ± 0.65		2.86 ± 0.55		4.45 ± 1.65	
Nongrassroots troop	3.24 ± 0.68		2.36 ± 0.63		2.90 ± 0.48		4.69 ± 1.48	

^*^p < 0.05.

SD, standard deviation.

### Correlation analysis

The analysis of correlations between each CMIS dimension and intention to stay is presented in Table 5. The path analysis was conducted according to the analysis of correlations between each of the 10 dimensions and intention to stay, the significant basic characteristic variables listed in Table 4, and a road map of the CMIS framework.

**Table 5:** Correlation of the continuous variables.

**Variables**	**1**	**2**	**3**	**4**	**5**	**6**	**7**	**8**	**9**	**10**
1. Manager characteristics	1									
2. Workload	−0.638**	1								
3. Career opportunities	0.613**	−0.673**	1							
4. Working environment	0.577**	−0.663**	0.698**	1						
5. Work organization	0.617**	−0.698**	0.687**	0.790**	1					
6. Coworker support	0.674**	−0.672**	0.545**	0.566**	0.667**	1				
7. Self-fulfillment	0.569**	−0.679**	0.599**	0.599**	0.649**	0.706**	1			
8. Job satisfaction	0.455**	−0.513**	0.460**	0.423**	0.486**	0.563**	0.607**	1		
9. Job stress	0.238**	−0.107	0.227**	0.262**	0.177**	0.096	0.039	0.105	1	
10. Institutional identification	0.556**	−0.625**	0.593**	0.548**	0.631**	0.688**	0.779**	0.599**	0.069	1
11. Intention to stay	0.254**	−0.316**	0.256**	0.226**	0.260**	0.364**	0.506**	0.421**	−0.179**	0.554**

^**^p < 0.01.

### Path analysis

**Figure 1:**
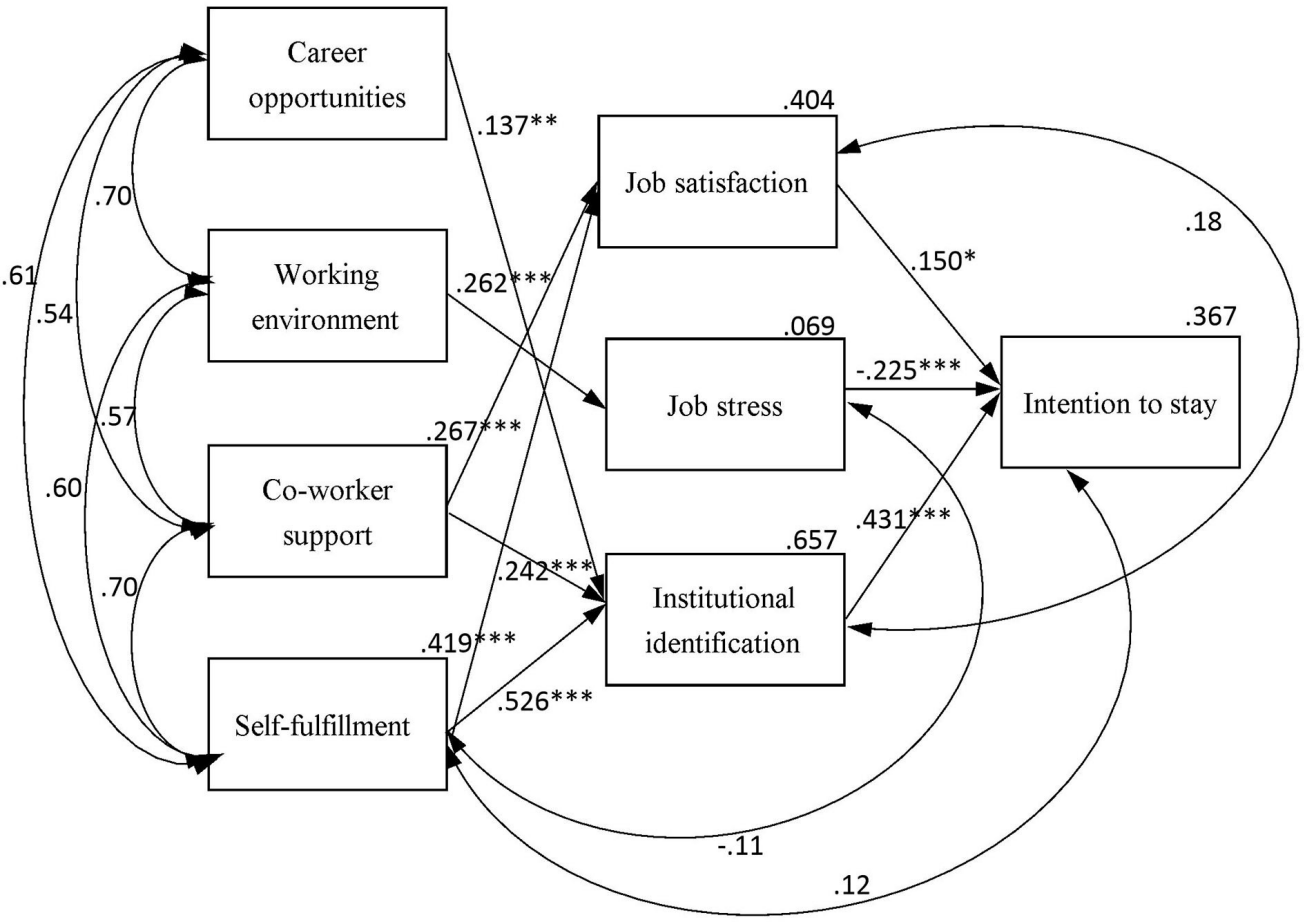
Path diagram of the conceptual model of intent to stay (CMIS) (path coefficients are indicated in the path diagram). *p < 0.05, **p < 0.01, ***p < 0.001.

Figure 1 presents the final model of the path analysis results. Table 6 presents the GIFs for the model, where χ^2^ (10) is 7.538 (*p* = 0.674), χ^2^/df is 0.754 (between 1 and 3), SRMR is 0.019 (<0.08), RMSEA is <0.001 (<0.05), GFI is 0.993 (>0.90), NFI is 0.993 (>0.90), non-normed fit index is 1.006 (>0.90), and comparative fit index is 1.000 (>0.95).

**Table 6:** Goodness-of-fit indices for the model.

**Fit index**	**Value**	**Recommended value**
*χ^2^*/df	0.754	1 < NC <3
*P*	0.674	>0.50
SRMR	0.019	<0.08
RMSEA	<0.001	<0.05
GFI	0.993	>0.90
NFI	0.993	>0.90
NNFI	1.006	>0.90
CFI	1.000	>0.95

df, Degree of freedom; SRMR, Standardized root mean square residual; RMSEA, Root mean square error of approximation; GFI, Goodness-of-fit index; NFI, Normed fit index; NNFI, Non-normed fit index; CFI, Comparative fit index.

Table 7 details the direct and indirect effects of CMIS variables on intention to stay, institutional identification, job stress, and job satisfaction. Institutional identification (β = 0.431, *p* < 0.001), job stress (β = −0.225, *p* < 0.001), and job satisfaction (β = 0.150, *p* = 0.014) all had a significant and direct effect on intention to stay. Self-fulfillment had a significant indirect effect on intention to stay (β = 0.290) through institutional identification and job satisfaction, whereas self-fulfillment had significant direct effect on institutional identification (β = 0.526, *p* < 0.001) and job satisfaction (β = 0.419, *p* < 0.001). Coworker support had a significant indirect effect on intention to stay (β = 0.144) through job satisfaction and institutional identification, whereas coworker support had significant direct effect on job satisfaction (β = 0.267, *p* < 0.001) and institutional identification (β = 0.242, *p* < 0.001). Working environment had a significant indirect effect on intention to stay (β = −0.059) through job stress, whereas working environment had a significant direct effect on job stress (β = 0.262, *p* < 0.001). In addition, career opportunities had a significant indirect effect on intention to stay (β = 0.059) through institutional identification, whereas career opportunities had significant direct effect on institutional identification (β = 0.137, *p* = 0.003).

**Table 7:** Path analysis result—direct and indirect effects.

**Dependent variable**	**Independent variable**	**Direct effect**	**Indirect effect**	**Total effect**
Intention to stay	Career opportunities		0.059	0.059
	Working environment		−0.059	−0.059
	Coworker support		0.144	0.144
	Self-fulfillment		0.290	0.290
	Job satisfaction	0.150		0.150
	Job stress	−0.225		−0.225
	Institutional identification	0.431		0.431
Institutional identification	Career opportunities	0.137		0.137
	Coworker support	0.242		0.242
	Self-fulfillment	0.526		0.526
Job stress	Working environment	0.262		0.262
Job satisfaction	Coworker support	0.267		0.267
	Self-fulfillment	0.419		0.419

## Discussion

This study revealed that the CMIS can be applied to analyze the factors that influence military smoking cessation counselors' intention to stay. Our research indicated that job satisfaction, job stress, and institutional identification can indirectly affect military smoking cessation counselors' intention to stay. Among these factors, institutional identification exerts the most significant effect, followed by job stress, whereas job satisfaction exerts the smallest effect. Career opportunities, coworker support, and self-fulfillment influence military smoking cessation counselors' intention to stay through institutional identification. Coworker support and self-fulfillment influence the military smoking cessation counselors' intention to stay through job satisfaction. Working environment influences the military smoking cessation counselors' intention to stay through job stress.

Military smoking cessation counselors' intention to stay is directly and positively influenced by institutional identification as an intervening variable, which is consistent with the results of previous studies (10, 16–18). According to the results of path analysis, among the effects of the three intervening variables on the smoking cessation counselors' intention to stay, that of institutional identification was the highest (β = 0.431). A related study that also used the CMIS and treated institutional identification as an intervening variable reported that the effect of institutional identification on the intention to stay was stronger among hospital nurses and psychosocial staff (β = 0.28 and 0.28, respectively) than among other hospital employees (β = 0.19 and 0.24 for physicians and administrative staff, respectively) (10). However, all of these values were much lower than the value obtained in this study (β = 0.431), reflecting our aforementioned conclusion that institutional identification exerted a strong effect on the military smoking cessation counselors' intention to stay. In Taiwan, the military smoking cessation counselors' work is a part-time endeavor; thus, they have opportunities to employ skills and abilities in addition to those employed in their principal job. Nevertheless, a sense of belonging and a sense of institutional identity exerted a stronger effect on the counselors' intention to stay than did job satisfaction and job stress. In addition, career opportunities, coworker support, and self-fulfillment all exerted a direct and positive effect on institutional identification, which is consistent with previous findings (19–23).

Military smoking cessation counselors' intention to stay is also positively influenced by job satisfaction as an intervening variable, and previous research has yielded similar results (9, 10, 24, 25). The path analysis revealed that among the effects of the three intervening variables, that of job satisfaction on intention to stay was the least (β = 0.150). However, prior researches that employed the CMIS and treated job satisfaction as an intervening variable reported that job satisfaction had the strongest effect on intention to stay among hospital physicians, laboratory staff, psychosocial staff, and critical care nurses (β = 0.24, 0.25, 0.35, and 0.308, respectively) (9, 10). A study reported that among part-time dental hygiene faculty members, job satisfaction was the strongest predictor of intention to stay (26). One potential explanation for this discrepancy is that the military prioritizes obedience, responsibility, and discipline. Job satisfaction may not be as central as institutional identification and job stress in informing military-affiliated professionals' intention to stay. Nevertheless, in this study, coworker support and self-fulfillment exerted a direct and positive effect on job satisfaction, which is consistent with previous findings (9, 10).

Military smoking cessation counselors' intention to stay is negatively influenced by job stress as an intervening variable, and previous research has yielded similar results (27, 28). Path analysis indicated that, among the three intervening variables, job stress exerts only the second most significant effect on smoking cessation counselors' intention to stay (β = −0.225). However, prior research that employed the CMIS and treated job stress as an intervening variable indicated that job stress exerted a negative effect on nurses' intention to stay in the hospital (β = −0.052) even smaller than that identified in this study (β = −0.225) (9). Although the military smoking cessation counselors' part-time job stress was not the strongest intervening variable, if part-time cessation counseling employment enhances the counselors' stress, it may lower their intention to stay. This suggests that job stress exerts a stronger effect on intention to stay among part-time employees than among full-time employees. Furthermore, working environment exerts a direct and positive effect on job stress, which is consistent with prior findings that decreasing part-time work reduces work stress (29). For military smoking cessation counselors, the need to improve their working environment, equipment, smoking cessation medications, and other associated consumables enhances work-related stress.

This study used the CMIS to explore the military smoking cessation counselors' intention to stay, and 36.7% of the variance in intent to stay among the smoking cessation counselors was explained using the model. This is comparable to the explanatory power of the CMIS reported in a study on willingness to stay among hospital employees of different occupations, which ranged from 23.6 to 36.0% (23.6, 26.1, 27.8, 31.4, and 36.0% for physicians, laboratory staff, administrative staff, nurses, and psychosocial staff, respectively) (10). Therefore, the CMIS can be used to predict military smoking cessation counselors' intention to stay.

In this study, the smoking cessation counselors' average score for intention to stay was 4.64 points (1–7 points). Studies have reported that the average willingness-to-stay scores of critical care nurses in intensive care units at urban hospitals, regular nurses employed in long-term care facilities, nursing assistants in nursing homes, and nursing assistants in long-term care facilities were 13.96 points (4–20 points), 3.5 points (1–5 points), 2.84 points (1–4 points), and 9.78 points (3–13 points), respectively, all of which are higher than our study population's average intention to stay (9, 30–32). These studies were conducted in the context of a nursing shortage and heavy workload, and the intention to stay was still higher than that of this study. This shows that the participants in the present study had low intention to stay. This low intention to stay may be attributable to the fact that the highest degrees of 61.3% of the participants in this study were in nonmedical fields or to the fact that smoking cessation counseling is part-time rather than full-time work (33).

## Limitation

This study has the following limitations: (1) We used online questionnaires to collect data from counselors, but we were unable to regulate the time it took for them to respond, which resulted in a lower sample recovery rate that may have led to the research findings failing to accurately reflect the actual situation. (2) Because the participants in this study were military smoking cessation counselors from 2016 to 2017, the results may not apply to all counselors; however, the findings may still have practical implications. (3) The participants' feelings were investigated using a self-administered questionnaire. When completing the questionnaire, a participant's responses may be influenced by their external environment, personal cognition, emotional state, attitude, and other factors, and participants may have doubts about and biased understandings of the items, resulting in errors in the research results. However, the questionnaire employed in this study exhibited high reliability and validity, indicating that the questionnaire's quality was sufficient. As much as possible, the survey can be conducted face-to-face to reduce bias due to the external environment. For instance, we can conduct questionnaires after the training sessions. (4) This was a cross-sectional study; thus, conclusions cannot be drawn regarding cause-and-effect relationships between the variables.

## Conclusion

This study verified that the CMIS can be used to predict military smoking cessation counselors' intention to stay. The results indicated that intention to stay was directly affected by job satisfaction, job stress, and institutional identification. Career opportunities indirectly affected intention to stay through institutional identification; the working environment indirectly affected intention to stay through job stress; and coworker support and self-fulfillment indirectly affected intention to stay through job satisfaction and institutional identification. The most effective strategy to improve smoking cessation counselors' intention to stay is to increase their institutional identification by encouraging military decision makers to implement policies designed to establish suitable reward and support systems. Policy makers in the military can establish a suitable reward based on military smoking cessation counselors' performance, such as how many smokers they helped successfully quit smoking, and award varying degrees of additional bonuses or gift certificates. Policy makers in the military can even more regularly praise the highly effective military smoking cessation counselors in public and award medals. In terms of promotion system, it is necessary to regularly and publicly commend those excellent military smoking cessation counselors in order to progress them to advanced status and give them more power and duty. In addition, future studies can assess the effect of smoking cessation counseling on smoking cessation rates and persistence.

## Data availability statement

The original contributions presented in the study are included in the article, further inquiries can be directed to the corresponding author.

## Ethics statement

The studies involving human participants were reviewed and approved by Institutional Review Board of the Tri-Service General Hospital, National Defense Medical Center. The patients/participants provided their written informed consent to participate in this study.

## Author contributions

Y-CL, Y-LC, SK, S-HC, F-GL, and C-HL designed the study and wrote the protocol. Y-CL, S-HC, C-YL, L-CY, F-YL, Y-TC, C-CW, and M-HL conducted literature searches and provided summaries of previous research studies. Y-LC, S-HC, and Y-CL conducted the statistical analysis. Y-CL wrote the first draft of the manuscript. All authors contributed to interpreting the results, read and agreed to the published version of the manuscript.

## Funding

This work was supported by the Ministry of National Defense-Medical Affairs Bureau (MAB-106-088), Taiwan, ROC.

## Conflict of interest

The authors declare that the research was conducted in the absence of any commercial or financial relationships that could be construed as a potential conflict of interest.

## Publisher's note

All claims expressed in this article are solely those of the authors and do not necessarily represent those of their affiliated organizations, or those of the publisher, the editors and the reviewers. Any product that may be evaluated in this article, or claim that may be made by its manufacturer, is not guaranteed or endorsed by the publisher.
